# The microbiome of the dinoflagellate *Prorocentrum cordatum* in laboratory culture and its changes at higher temperatures

**DOI:** 10.3389/fmicb.2022.952238

**Published:** 2022-09-28

**Authors:** Selene Sanchez-Garcia, Hui Wang, Irene Wagner-Döbler

**Affiliations:** Institute of Microbiology, Technical University of Braunschweig, Braunschweig, Germany

**Keywords:** dinoflagellate, *Prorocentrum cordatum*, temperature, 16S/18S ribosomal RNA gene analysis, microbial communities, growth rate

## Abstract

In the ocean, phytoplankton are dependent on communities of bacteria living in the phycosphere, a hot spot of metabolic and genetic exchange. Many types of interactions between phytoplankton and phycosphere bacteria have been shown, but it is unclear if the microbial communities associated with microalgae strains in culture collections are beneficial or harmful to the host strain. Here, we studied the microbial communities associated with four strains of the dinoflagellate *Prorocentrum cordatum* that had been isolated from distant geographical locations and maintained in culture collection for hundreds of generations. Community composition was determined by 16S rRNA gene amplicon sequencing. The dinoflagellate host strain was the strongest parameter separating communities, while growth phase, lifestyle (particle-attached versus free-living) and temperature had only a modulating effect. Although the strains had been isolated from distant locations in the Atlantic and Pacific Ocean, 14 ASVs were shared among all strains, the most abundant ones being *Gilvibacter, Marivita*, uncultivated Rhodobacteraceae, *Marinobacter*, *Hyphomonadaceae*, *Cupriavidus*, *Variovorax,* and *Paucibacter*. Adaptation to higher temperatures resulted in specific changes in each phycosphere microbiome, including increased abundance of rare community members. We then compared the growth of the four xenic cultures to that of the axenic *P. cordatum* CCMP1329. At 20°C, growth of the xenic cultures was similar or slower than that of CCMP1329. At 26°C, all four xenic cultures experienced a death phase, while the axenic culture stably remained in the stationary phase. At 30°C, only two of the xenic cultures were able to grow. A shift of dinoflagellate metabolism from autotrophy to mixotrophy and competition between dinoflagellate and bacteria for limiting nutrients, including essential vitamins, may contribute to these differences in growth patterns.

## Introduction

Marine phytoplankton, although microscopic, accounts for about half of the world’s primary production (Field et al., 1998; Falkowski et al., 2004). About half of the carbon fixed by marine phytoplankton is excreted and is consumed by heterotrophic bacteria. The interactions between these two groups strongly influence carbon and nutrient cycling, regulate the productivity and stability of aquatic food chains and affect ocean–atmosphere fluxes (Azam and Malfatti, 2007). Phytoplankton and bacterial community composition have been demonstrated to stably re-occur from year to year (Needham and Fuhrman, 2016) suggesting highly specific interactions between them. Spring microalgae blooms have also shown recurrent patterns during several years of tracking (Teeling et al., 2016) emphasizing the specificity of the relationship between phytoplankton species and their bacterial communities. Interactions between phytoplankton and bacteria occur in a microscale level, within the volume of water closely surrounding the algal cell (Seymour et al., 2017). The “phycosphere,” a name analogous to the “rhizosphere” in terrestrial plants, is a diffusion limited layer that surrounds phytoplankton cells, and it is characterized by high concentrations of organic molecules relative to bulk seawater and close physical contact between bacteria and phytoplankton host, enhancing the potential for metabolic exchange and interactions (Seymour et al., 2017). Bacteria colonize the phycosphere by random encounter with the phytoplankton cells, by using chemotaxis or *via* vertical transmission, and they are found as intracellular symbionts, attached to the phytoplankton surface or free living (Seymour et al., 2017). Attached and free-living bacterial communities often show significant taxonomic differences (Grossart et al., 2005; Park et al., 2017).

The establishment of microalgae isolates in laboratory cultures is essential in order to study their physiology, biochemistry and cellular biology, and it usually includes the phycosphere microbiome that was present in the drop of water and on the surface of the algal cells at the time of isolation. L1 medium (enriched F/2 medium; Guillard and Hargraves, 1993), which is routinely used for marine phytoplankton in culture collections around the world, contains salts, trace elements and the three vitamins thiamine (B1), biotin (B7), and cobalamin (B12) for which a large fraction of microalgae are auxotrophic (Croft et al., 2006; Helliwell, 2017). Microalgae forming harmful blooms (HABs) for example are dominated by species that require B1 and B12 (Tang et al., 2010). In the ocean, these vitamins can be provided by phycosphere bacteria (Croft et al., 2005; Wagner-Döbler et al., 2010).

By repeated washing and treatment with antibiotics, bacteria can be removed from the phycosphere and the microalgal strain can then be cultivated without accompanying bacteria, a condition which is called “axenic.” Many phototrophic microalgae can be maintained in such a way on L1 medium, indicating that all they really needed from the bacteria were the vitamins. Examples of axenic microalgae cultures that can be obtained from the NCMA Bigelow culture collection are the haptophyte *Prymnesium parvum,* the chlorophyte *Tetraselmis suecica* and the diatom *Thalassiosira oceanica.*

However, even in the presence of excess vitamins many algae still maintain a heterotrophic bacteria community, which is entirely dependent on nutrients excreted by the algae and can be very difficult to remove. For example, some strains of the dinoflagellates *Karenia brevis* and *Gymnodinium catenatum* are unable to grow axenically (Monroe and Van Dolah, 2008; Steidinger, 2009; Bolch et al., 2011). There are controversial reports about the impact of the phycosphere bacteria on the algae. Growth of various marine diatom species was stimulated by a *Sulfitobacter* strain that synthesized the algal growth hormone indole-3-acetic acid from tryptophan, and since both of these compounds are infochemicals widely produced in coastal areas this type of interaction might be ubiquitous (Amin et al., 2015). The toxic dinoflagellate *Gymnodinium catenatum* showed a reduced growth rate and an increased death rate when co-cultivated with a Roseobacter, but not when a mixed inoculum or Gammaproteobacteria was used for co-cultivation (Bolch et al., 2017). Degradation products of the algal metabolite DMSP, which is one of the main sulfur and carbon sources used by phycosphere bacteria, turned a *Sulfitobacter* strain into a pathogen (Barak-Gavish et al., 2018). The roseobacter species *Dinoroseobacter shibae* can provide vitamin B12 to the dinoflagellate *Prorocentrum cordatum* during exponential growth but kills the dinoflagellate at a later growth stage (Wang et al., 2014). Such a “Jekyll-and-Hyde” interaction has also been postulated for *Emiliania huxleyi* where the associated *Phaeobacter gallaeciensis* strain synthesizes Roseobacticides from microalgal degradation products (Seyedsayamdost et al., 2011). For *Prorocentrum lima* it has often been suspected that the associated bacteria influence toxin production of the dinoflagellate (Tarazona-Janampa et al., 2020). The close relationship between bacteria and their algae host is not specific for phytoplankton. Members of *Rhodobacteraceae* and *Flavobacteriaceae* can completely recover the growth and morphogenesis of axenic green macro-algae *Ulva mutabilis* cultures, providing a novel mode of action for bacteria-induced algal development (Wichard, 2022).

Here we studied the phycosphere microbiome of the dinoflagellate *Prorocentrum cordatum.* This globally abundant, invasive, potentially toxic dinoflagellate was previously named *P. minimum* but a taxonomic re-analysis resulted in its renaming and assignment to *P. cordatum* (Velikova and Larsen, 1999; Khanaychenko et al., 2019). It is a cosmopolitan harmful dinoflagellate that causes blooms at increasing frequency, duration and magnitude (Hallegraeff, 1993; Heil et al., 2005; Zhang et al., 2021). *P. cordatum* was initially found in the Krasnovodsky Bay of the Caspian Sea, and has expanded its habitat to coastal ecosystems worldwide (Khanaychenko et al., 2019), including the North Atlantic, where it is now regularly detected at the Helgoland long-term monitoring station (Seebens et al., 2016). During the past decades the frequency and intensity of its blooms have been increasing (Zhang et al., 2021). The dinoflagellate has proven to be highly physiologically flexible under diverse environmental conditions, such as light, salinity and temperature (Grzebyk and Berland, 1996; Zhang et al., 2021).

*P. cordatum* has repeatedly been associated with toxic microalgal blooms (Heil et al., 2005; Khanaychenko et al., 2019). *P. cordatum* strains from French Mediterranean sites have been reported to contain a water-soluble neurotoxic component which killed mice within minutes at high doses (Grzebyk et al., 1997; Denardou-Queneherve et al., 1999). Recently, a *P. cordatum* bloom has been associated with the accumulation of the neurotoxin tetrodotoxin in shellfish in the Mediterranean Sea, and cultured strains showed sodium channel blockage activity at higher temperatures (26°C compared to 18°C; Rodríguez et al., 2017). However, a toxic compound has not yet been identified.

Due to its ability to colonize and bloom in contrasting geographic locations, the dinoflagellate *P. cordatum* is mainly researched for its physiological plasticity; however, little is known about the bacterial community that lives with this host. In this study, we examined the composition of the microbial communities associated with four different, xenic strains of *P. cordatum* which were isolated from marine habitats around the world more than 17 years ago and maintained in L1 medium ever since. We asked if a core microbial community of *P. cordatum* exists, or if each dinoflagellate strain hosts a different microbiome. *P. cordatum* has been reported to grow with a maximum growth rate between 18°C and 26.5°C, and to still grow, slowly, up to 31°C (Grzebyk and Berland, 1996). The different *P. cordatum* strains were grown at 20°C since it was the maintenance temperature of strain CCMP1329 (axenic). An increase to 26°C is still within the optimal temperature range, but differences in growth rate and microbiome composition might be detectable depending on strain origin. 30°C is ecologically relevant as it is already encountered in summer, influencing dinoflagellate blooms (Tester et al., 2020; Owen et al., 2022). Furthermore, in the future ocean, the time periods where temperatures between 26°C and 30°C are encountered by marine dinoflagellates will be prolonged (Alexander et al., 2018). Therefore, we asked how these two temperatures will affect the phycosphere community composition. To this end, we transferred the cultures from their maintenance temperature (20°C) to 26°C or 30°C and analyzed the shifts in community composition. Cultures were sampled at exponential and stationary growth phase, and since free living and particle attached communities might have different roles in the phycosphere, they were analyzed separately. Finally, we asked if there were differences in the growth of the four *P. cordatum* xenic strains compared to that of the axenic isolate (CCMP1329) when cultures were grown at these three temperatures.

## Materials and methods

### Cultivation of *Prorocentrum cordatum* strains

The xenic strains of *P. cordatum* CCMP697, CCMP698, CCMP1529, and CCMP2811 and the axenic strain CCMP1329 were obtained from the Provasoli-Guillard National Center for Marine Algae and Microbiota (NCMA, formerly the Provasoli-Guillard National Center for Culture of Marine Phytoplankton, CCMP). Date and place of isolation for each strain can be found in Table 1. After arrival at our lab, the *P. cordatum* isolates were cultured for at least 4 transfers (around 2 months) at 20°C to acclimatize them to our culture conditions before growing them at 26°C and 30°C.

**Table 1:** Isolation and deposit year, collection site, known temperature range, and maintenance temperature at NCMA of *Prorocentrum cordatum* strains investigated in this study.

Strain	Isolation year	Deposit year	Collection site	Known temperature range	Maintenance temperature at NCMA
CCMP697	1979	1985	North Sea 59.3° N 10.36° E	11°C–16°C	14°C
CCMP698	1988	1989	Gulf of Maine 43.9106° N 69.9384° W	11°C–22°C	14°C
CCMP1529	1991	1992	South Pacific 2.6667° S 82.7167° W	18°C–22°C	20°C
CCMP2811	2005	2007	Gulf of Mexico 27.3335° N 82.583° W	18°C–22°C	20°C
CCMP1329^*^	NA	1958	North Atlantic 40.6667° N 73.25° W	20°C–26°C	20°C

* Axenic.

The five *P. cordatum* strains were cultivated in incubators set to 20°C, 26°C, and 30°C as previously described (Wang et al., 2014). Briefly, the cultures were cultivated in L1 medium prepared according to (Guillard and Hargraves, 1993) with the modifications that synthetic ocean water (Sunda et al., 2005) was used instead of natural seawater, and Na_2_SiO_3_⋅9H_2_O was omitted, since *P. cordatum* does not require silica. A 12:12 h light–dark cycle with light intensity of about 40 μmol quanta m^−2^ s^−1^was set on the three incubators. The cultures were maintained by transferring 10% of the culture volume at late exponential phase to fresh medium, which corresponded to every 2 weeks for the cultures grown at 20°C, every 10 days for the cultures grown at 26°C and 1 week for the cultures grown at 30°C. The dinoflagellate cultures were maintained at each temperature for at least four transfers before samples for MiSeq sequencing were collected. All experimental work and dinoflagellate transfers were performed under a laminar flow hood using sterile conditions. Lack of contaminating bacteria in strain CCMP1329 was checked by routinely streaking aliquots on LB and Difco marine agar 2216 (MB) plates and by occasionally performing 16S PCR assay.

Growth of *P. cordatum* was followed by cell counting by flow cytometer. Before cell counting, 1 ml from each sample was taken in the light period and fixed with 25% glutaraldehyde to a final concentration of 2% for about 20 min at room temperature (Wang et al., 2014). Growth of strains CCMP2811, CCMP1529, CCMP1329, and CCMP698 at 20°C, and strains CCMP2811 and CCMP1529 at 26°C was followed by using a BD FACS Canto flow cytometer (BD Biosciences, San Jose, CA, United States), according to the methods described previously (Wang et al., 2014; Mansky et al., 2022). Due to this cytometer being out of service, the rest of the growth curves were performed by a MACSQuant X flow cytometer (Milteny Biotec, Bergisch Gladbach, Germany). A 90 well microtiter plate was used for measurements. Wells were filled with 200 μl of sample. Each sample was measured twice, and the average was used for further analysis. Chlorophyll a was excited with a 488 nm excitation laser line and emitted at 695 nm (far red). The intake volume was set at 80 μl using a medium flow rate of 50 μl/min. The cytometers settings were as follows: forward scatter (FSC) = 300, far red fluorescence (PerCP-Cy5.5) = 300. Given that there was discrepancy between the counts of events/ml between both cytometers, the cell density between cultures could not be compared. However, since growth rate and doubling time are independent of the absolute number of cells a direct comparison of these parameters between strains and temperatures is possible.

For each strain, three biological replicates were measured every 2–3 days for 24–26 days at 20°C and 26°C and for 15 days at 30°C. This was done for 3 consecutive cultures, to obtained three independent growth curves.

Cell concentrations of biological replicates were averaged, and the three growth curves per strain at each temperature were plotted against time and a generalized additive model (GAM) was fitted to the data for each strain at each temperature using the function geom_smooth(method = “gam”) in the program R. GAMs were used due to their ability to fit nonlinear and/or non-monotonic functions. The specific growth rate in the exponential growth phase, μ_exp_ (day^−1^) was calculated according to the following equation:


$$r=\frac{\ln\left(\frac{N_{t}}{N_{0}}\right)}{\Delta t}=\frac{lnN_{t}-lnN_{0}}{\Delta t}$$

where N_0_ and N_t_ are cells/ml at the beginning and end of the exponential growth phase from time t_0_ to t. The concentration (cell/ml) per day values were obtained from the GAM model fitted to each *P. cordatum* strain at each temperature. The growth rate function (in the program R), available at https://scholar.princeton.edu/botsteinlab/protocols was used with a coefficient of determination of the linear fit R^2^ > 0.9 to identify the region of exponential growth. All models had a *p*-value < 0.001. Dividing the specific growth rate by the natural log of 2 (0.6931) will give doublings per day (Andersen et al., 2005).

### Sampling

Experimental cultures were prepared in a volume of 100 ml in triplicate. Exponential and stationary phase were analyzed in separate flasks inoculated from the same pre-culture. Strains CCMP698 and CCMP1529 did not grow at 30°C. To obtain the particle attached (PA) bacterial communities, 50 ml of *P. cordatum* culture were filtered through 3 μm polycarbonate membrane filters (Merck Millipore, Billerica, MA, United States; diameter: 47 mm) without pressure. Cells retained on the filter were washed three times with sterilized artificial seawater. The filtrate was passed through a 0.22 μm polycarbonate membrane filter (Merck, Millipore; diameter: 47 mm) without pressure to retain the free-living (FL) bacteria. Filters were stored at −80°C until DNA extraction was performed.

### DNA extraction and sequencing

Filters were placed into 5 ml PowerWater DNA Bead tubes (Qiagen, Germany) and DNA was isolated from the cells on the filters using the DNeasy PowerWater Kit (Qiagen, Germany) following the manufacturer’s instructions. A bead mill homogenizer (VWR, United States), was used for 1 min at speed 6 m/s for the cell lysing step. DNA concentration was measured using Qubit 4 (Invitrogen, United States) and DNA was stored at −20°C until preparation of the 16S sequencing library.

The 16S rRNA gene hypervariable region V4-V5 was amplified using the universal 16S/18S primer set 515-Y F and 926R (Parada et al., 2016). Amplification and library preparation was done according to Yeh et al. (2021). For DNA samples with concentrations below 0.5 ng, three independent PCR runs were performed and pooled afterwards to increase final DNA concentration. Samples where still DNA was not detected after pooling were left out of the analysis. For each sequencing run, mock communities for 16S gene sequences of common marine bacteria (Parada et al., 2016) as well as PCR negative controls were added to the pooled amplicon library. Pools were sequenced on an Illumina MiSeq platform using PE300 chemistry. The raw data is deposited at NCBI (BioProject accession number PRJNA831420).

### Sequence analysis

All raw data were analyzed using QIIME2 and the DADA2 denoising algorithm (Callahan et al., 2016) described in detail in (Yeh et al., 2021). Briefly, sequencing results were demultiplexed, and primer sequences were trimmed using cutadapt allowing 1 mismatch within the primer sequences. Sequences were then separated into 16S and 18S reads using the bbsplit package. Dinoflagellate reads were separated from the library at this step. The 16S reads were further analyzed using DADA2 QIIME2 version 2019.4 (Bolyen et al., 2019). Forward reads were trimmed at 250 bp and reverse reads at 220 bp. Subsequently, sequences were denoised, and forward and reverse reads were merged allowing no mismatch and requiring an overlap of at least 20 bp. ASVs were defined allowing one mismatch and chimeras were removed using the q2-dada2 plugin algorithm (Callahan et al., 2016). Taxonomy was assigned with the classify-sklearn plugin against the SILVA 132 database that was partitioned to the amplicon region (Bokulich et al., 2018). ASVs classified as chloroplast were filtered out. The scripts for the bioinformatics pipeline are available at http://github.com/jcmcnch/eASV-pipeline-for-515Y-926R. All steps were executed within a standardized CONDA environment to allow reproducibility of results.

### Statistical analysis

All data analyses were performed with the program R version 4.2.0 (R Development Core Team, 2019) using the packages ggplot2 (Wickham, 2009), phyloseq (McMurdie and Holmes, 2013) and vegan (Oksanen et al., 2017). Prior to statistical analysis, samples with less than 2000 counts were removed from the analysis as well as ASVs with less than 5 counts in total and ASVs linked to contamination (Supplementary File 1). Considering that there are triplicate samples for each condition, ASVs not found in at least two samples with a minimum of 3 reads were also filtered out. Triplicate samples were combined if necessary using the function: merge_samples_mean() from (Denef et al., 2016). The combined dataset was comprised of four conditions (exponential-FL, exponential-PA, stationary-FL, stationary-PA) for each strain at each temperature.

For alpha diversity analysis, reads were rarefied to 7,000 counts per sample (the sample with the smallest read count of all samples). Shannon diversity and richness were calculated with the rarefied data using the package vegan. The Kruskal–Wallis test with adjusted Benjamini–Hochberg value of *p*-values was performed to evaluate differences in the bacterial communities between *P. cordatum* strains and the Wilcoxon test was used to evaluate differences between temperature, lifestyle and growth phase within the bacterial communities of each strain. To investigate the beta-diversity, relative abundance per sample was calculated and square-root transformed (Hellinger transformation). Bray–Curtis dissimilarity was calculated for the transformed data and the matrix was explored by non-metric Multi-Dimensional Scaling (NMDS) plots using the function metaMDS from the vegan package. For testing significant differences in the beta diversity between *a priori* defined groups (strains, temperature, size fraction, growth phase) a Permutational Analysis of the Variance (PERMANOVA) on the Bray–Curtis dissimilarity matrices with 10,000 permutations using the adonis() function from the vegan package was performed.

In order to properly handle the large number of zeros inherent in amplicon sequencing data, the amplicon count matrix was preprocessed using the function feature_table_preprocess() with default settings from the ANCOM-II (analysis of compositions of microbiomes) R package, which classifies zeros based on the geometric mean of ASVs within the specimen. A new count matrix was obtained where zeros were classified according to (Kaul et al., 2017) as “structural zeros” for ASVs that are not expected to be in one dinoflagellate strain but can be present in others and “sampling zeros” for ASVs that should be present but due to low abundance/sequencing depth could not be detected. After processing, the ASV counts were log transformed and displayed in heatmaps. ASVs with a log2 fold change ≥ 1 and a *p*-value < 0.05 were considered as having a significant change in abundance.

## Results

### Sequencing data

The microbial communities of the dinoflagellate isolates were characterized using high-throughput 16S gene amplicon sequencing. There were no archaeal reads found in any of the samples. After preprocessing and filtering a total of 2,441,113 reads were obtained, comprising 127 ASVs in 111 samples (Supplementary File 2). Two samples (698–0.22um-exp-3a and 2,811–30.20C-0.22um-exp-3c) were outliers and therefore not used for the analysis (Supplementary File 1). From the 127 identified ASVs, 29 remained unassigned at genus level using SILVA 132 classification. From these unassigned ASVs, 16 were assigned to the family *Rhodobacteraceae.*

### Similarity between the microbial communities of *Prorocentrum cordatum* strains

**Figure 1:**
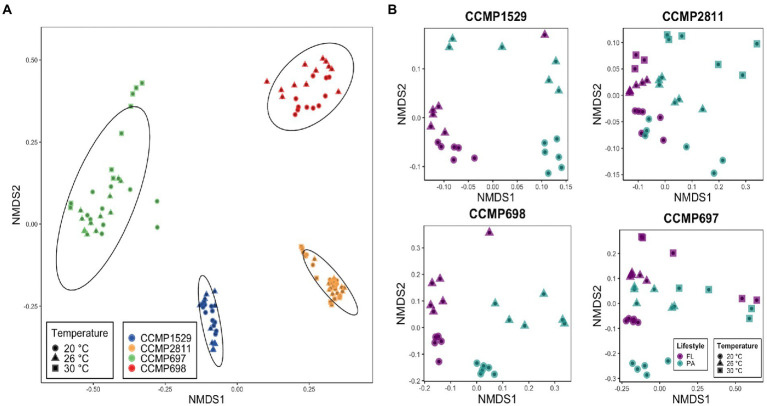
Non-metric multidimensional scaling (NMDS) plots analyzed with Bray–Curtis distance matrices based on Hellinger transformed ASV counts. **(A)** Dissimilarities across *Prorocentrum cordatum* strains’ bacterial communities. Ellipses represent 95% confidence interval of the standard error of the points from the centroid of the clusters. **(B)** Dissimilarities within the bacterial communities of each *P. cordatum* strains. Stress for all ordinations: <0.2.

To investigate if the composition of the bacterial communities associated with *P. cordatum* was similar across isolates or specific to each strain, beta-diversity was determined using an NMDS analysis based on Bray–Curtis dissimilarity (Figure 1A). The NMDS plot revealed distinct clustering of the samples from each *P. cordatum* strain regardless of temperature, lifestyle or growth phase. Strain CCMP697 showed the largest within strain variation, with three of the 30°C samples and two from 20°C being clearly distant from the centroid. PERMANOVA analysis based on the Bray–Curtis matrix confirmed that the four strains had significantly different bacterial communities (*R*^2^ = 0.82, *p* < 0.001). When analyzing the microbiome of each dinoflagellate strain individually, samples clustered according to lifestyle and temperature (Figure 1B). The three parameters lifestyle, temperature and growth phase had a significant impact on the composition of the bacterial communities of each *P. cordatum* strain. Lifestyle and temperature explained most of the variability, while the influence of growth phase was minor (Table 2).

**Table 2:** Influence of temperature, lifestyle and growth phase on beta-diversity of bacterial communities of four *Prorocentrum cordatum* strains.

	CCMP1529		CCMP2811		CCMP698		CCMP697	
	*R^2^*	*Pseudo-F*	*R^2^*	*Pseudo-F*	*R^2^*	*Pseudo-F*	*R^2^*	*Pseudo-F*
Temperature	0.217^***^	12.25	0.183^***^	10.88	0.247^***^	14.36	0.327^***^	18.82
Lifestyle	0.344^***^	19.45	0.244^***^	14.49	0.349^***^	20.15	0.102^**^	5.91
Growth phase	0.084^**^	4.74	0.083^**^	4.95	0.072^**^	4.17	0.100^**^	5.76

Permutational multivariate analysis of variance (PERMANOVA) on Bray–Curtis dissimilarity of Hellinger transformed ASV counts of the bacterial communities of each *P. cordatum* strain was performed. Factors are temperature (20°C, 26°C, 30°C), lifestyle (particle attached, free-living) and growth phase (exponential and stationary growth phase).

** *p* < 0.01;

*** *p* < 0.001.

### Alpha-diversity of the microbial communities of *Prorocentrum cordatum* strains

**Figure 2:**
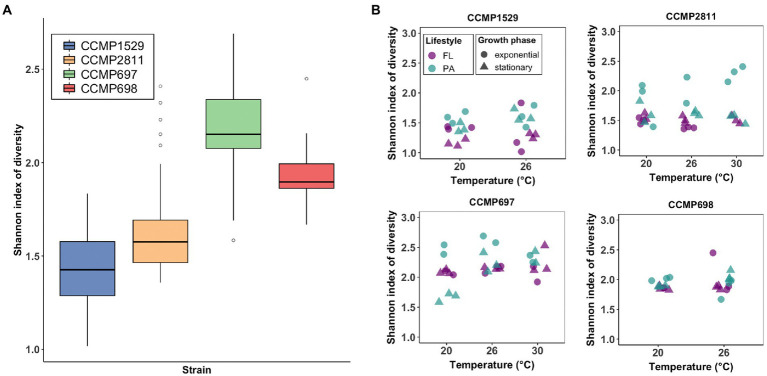
Analysis of Alpha diversity of the bacterial communities of *Prorocentrum cordatum* measured by mean Shannon diversity index. **(A)** Total Shannon diversity index of the bacterial communities. All samples were grouped by strain and are indicated by color. The solid black lines indicate medians, and the lower and upper bounds of the box represent the 25% and 75% quartiles. Outliers are indicated as open circles and represent samples falling outside the 10% and 90% quartiles. **(B)** The Shannon diversity between temperature (x-axis), lifestyles (color) and growth phase (shape) was analyzed for the bacterial communities of each *P. cordatum* strain.

The Shannon index of diversity was significantly different between all four communities (Kruskal–Wallis, *p* < 0.001; Figure 2A). Strain CCMP697 showed the highest diversity (2.18 ± 0.25), while the lowest was found in strain CCMP1529 (1.43 ± 0.21). It was then analyzed if diversity of the bacterial communities of each *P. cordatum* strain was influenced by temperature, lifestyle, and growth phase (Figure 2B). Shannon diversity at 30°C was significantly higher than in the other two temperatures for strain CCMP697. For the rest of the strains there was no significant difference in Shannon diversity between the three temperatures. Particle attached communities were significantly more diverse than free-living ones, except for the microbiome of strain CCMP697. There was no significant difference in Shannon diversity regarding growth phase for any of the strains. When analyzing richness, similar results were found (Supplementary Figure 1A), except that strain CCMP698 had a significantly richer bacterial community in its exponential phase compared to stationary phase (Supplementary Figure 1B).

### Composition of the bacterial communities associated with *Prorocentrum cordatum*

**Figure 3:**
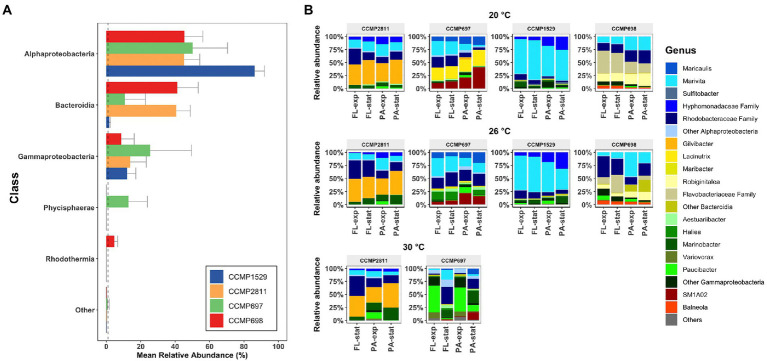
Composition of the bacterial communities of *Prorocentrum cordatum* xenic cultures. **(A)** Mean relative abundance of the bacteria (Class level) found in each *P. cordatum* strain. ASVs with an overall abundance below 1% are listed as “Others.” Dashed line indicates the 1% threshold. **(B)** Mean relative abundance of bacterial genera found in each *P. cordatum* strain at each temperature. Samples are grouped by triplicates and listed by lifestyle and growth phase. Colors represent bacterial genus level, where shades of blue correspond to genera from the class of Alphaproteobacteria, shades of yellow to Bacteroidia, shades of greens to Gammaproteobacteria, red to Phycisphaerae, and orange to Rhodothermia.

The microbial communities of the four *P. cordatum* strains were comprised predominantly of bacteria from the classes Alphaproteobacteria, Gammaproteobacteria, Bacteroidia, Phycisphaerae, and Rhodothermia (Figure 3A). Phycisphaerae were only found in the microbiome of strain CCMP697, mainly in its PA community (19.6% ± 11.9%) and Rhodothermia were only found in the microbiome of strain CCMP698 (4.56% ± 2.06%). The proportions of the three dominant classes varied considerably between the four *Prorocentrum* isolates. For example, more than 80% of strain CCMP1529 microbiome belonged to the Alphaproteobacteria, 12% to Gammaproteobacteria and just 1.7% to Bacteroidia. On the other hand, strains CCMP698 and CCMP2811 had very similar proportions of Alphaproteobacteria (45%) and Bacteroidia (41%). Gammaproteobacteria comprised between 8.7% (CCMP698) and 25% (CCMP697) of the microbiota.

The taxa dominating the communities associated with the four *P. cordatum* strains were different for each isolate and changed with increased temperature (Figure 3B). Within the Alphaproteobacteria, uncultured *Rhodobacteraceae* sequences were found across all strains at all three temperatures at high abundance, with ASV116 comprising 15.05% of all reads (Supplementary File 2). This ASV, which was assigned as uncultured *Rhodobacteraceae* by the SILVA 132 classification, was later assigned as *Marivita* after using the NCBI BLAST function. This *Marivita* ASV116 was the most abundant ASV for strain CCMP1529 at both temperatures (63.72% ± 7.5% at 20°C and 56.9% ± 13.45% at 26°C). It was also the most abundant ASV of strain CCMP697 microbiome at 26°C (24 ± 8%) but was absent in the bacterial communities of the other two *P. cordatum* strains. Interestingly, another ASV assigned as *Marivita* (ASV115), was abundant in the two other strains, namely CCMP698 and CCMP2811, at all temperatures. These two *Marivita* ASVs most likely represent different species of the genus *Marivita* with different ecological niches. Other dominant Alphaproteobacteria were uncultured *Hyphomonadaceae* (strains CCMP1529 and CCMP2811) and *Maricaulis* (strain CCMP697). *Gilvibacter* ASV23 accounted for all the Bacteroidia sequences of strain CCMP2811. This was also the most abundant ASV in the bacterial communities of strain CCMP2811 at all three temperatures (40.4% ± 1.7%). At 20°C, *Lacinutrix* was the predominant Bacteroidia genus in the microbiome of strain CCMP697 and decreased significantly at 26°C and 30°C. Strain CCMP698 had mainly *Robiginitalea* and uncultured Flavobacteriaceae sequences, and at 26°C the proportion of other Bacteroidia ASVs increased. Even though Gammaproteobacteria were found at much lower relative abundance than Alphaproteobacteria or Bacteroidia, they were the most diverse class across all *P. cordatum* strains (Supplementary File 3). Interestingly, *Paucibacter* increased drastically in the exponential phase of strains CCMP697 and CCMP2811 at 30°C. For the class Physisphaerae, found in higher abundance in strain CCMP697, only sequences matching the group SM1A02 were found, and the genus *Balneola* from the class Rhodothermia was unique in strain CCMP698.

### Unique and shared ASVs in the *Prorocentrum cordatum* cultures

**Figure 4:**
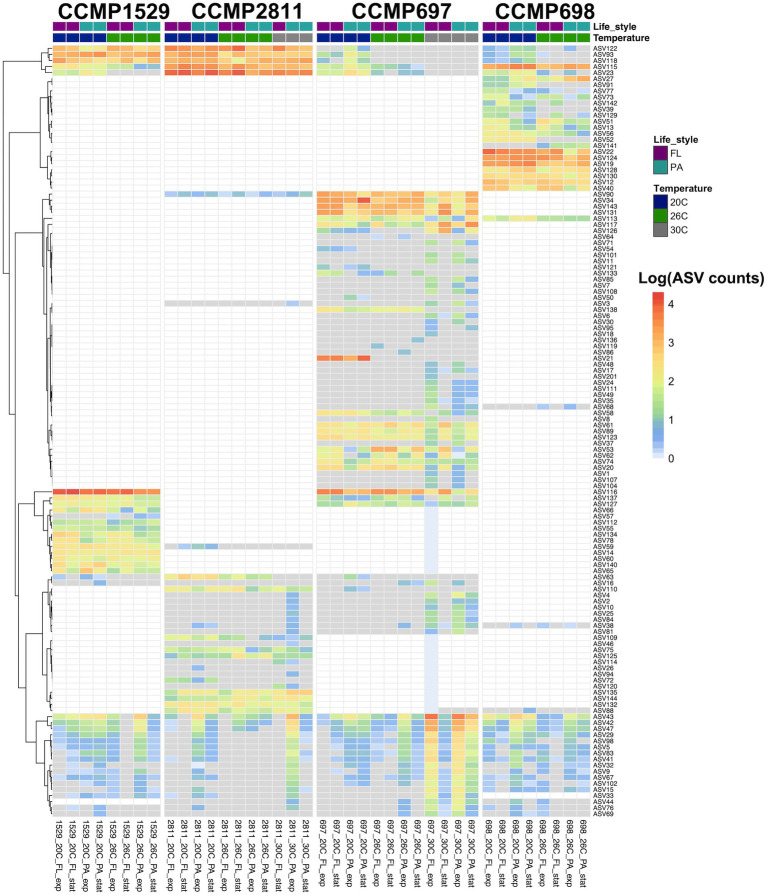
Abundance of all ASVs detected in four xenic strains of *Prorocentrum cordatum*. at different conditions and temperatures. The heatmap shows the hierarchical clustering of ASVs based on their abundances, expressed as log(1 + x). After AMCOM-II preprocessing, white squares correspond to “structural zeros” (lack of ASVs) and gray squares to “sampling zeros” (zeros due to abundance below detection limit/sequencing depth; Kaul et al., 2017). The average AVS abundance was calculated from two or three biological replicates for each strain at each condition, where the colors in the first row indicate lifestyle and the ones in the second-row temperature.

Each of the four *P. cordatum* strains had a distinct phycosphere community of ASVs which were only found in this specific strain (unique microbiome; Supplementary File 4), as well as ASVs shared among all four strains of *P. cordatum* (core microbiome). Figure 4 and Supplementary Figure 2 show all ASVs detected in the four strains and their log transformed abundance in each strain and condition.

From 84 ASVs found in the bacterial community of strain CCMP697, 45 were specific for this dinoflagellate strain. They belonged to seven different bacterial classes, in agreement with strain CCMP697 showing the highest bacterial diversity from the four *P. cordatum* isolates. Strain CCMP698 had 19 unique ASVs (from a total of 44), belonging to the classes Alphaproteobacteria, Gammaproteobacteria, Bacteroidia, and Rhodothermia. Strains CCMP1529 and CCMP2811 had just 10 and 12 unique ASVs out of a total of 37 and 48, respectively. The unique ASVs in strain CCMP1529 belonged to the three most dominant classes, while all the unique ASVs of strain CCMP2811 belonged to the Alphaproteobacteria.

**Figure 5:**
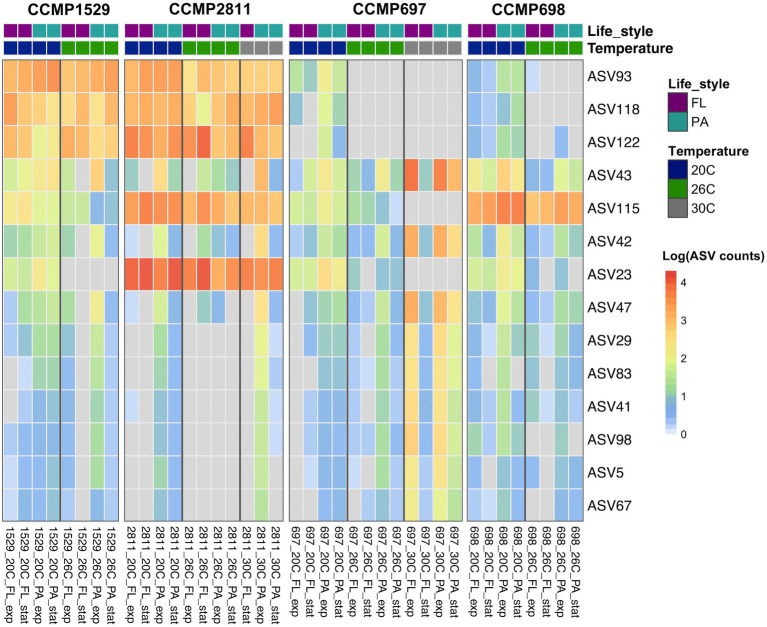
Core microbiome of *Prorocentrum cordatum*. Heatmap showing the abundance of the ASVs detected in all four xenic strains of *P. cordatum* at different conditions and temperatures. See Figure 4 for details of calculations. ASV rows are sorted by abundance.

Despite the clearly distinct phycosphere communities of the four strains of *P. cordatum*, we found ASVs that were shared between two or even all four dinoflagellate strains. Three ASVs (ASV116, ASV137, ASV127) were shared between CCMP1529 and CCMP697, and ASV113 was found in both CCMP697 and CCMP698 phycosphere communities. We found 14 ASVs that were shared among all *P. cordatum* strains and thus form a core community (Table 3). Figure 5 shows the abundances of these 14 ASVs at all conditions. Seven of them belonged to the most abundant ASVs in our study (ASV23, ASV115, ASV122, ASV118, ASV93, ASV42, ASV43, ASV47). They were among the 24 ASVs that together accounted for 92% of all reads. The other seven are rare members of the phycosphere microbiome.

**Table 3:** Core community of 14 ASVs shared between all four strains of *Prorocentrum cordatum.*

ASV No.	Phylum	Class	Order	Family	Genus
ASV5	Actinobacteria	Actinobacteria	Corynebacteriales	Nocardiaceae	*Rhodococcus*
ASV23	Bacteroidetes	Bacteroidia	Flavobacteriales	Flavobacteriaceae	*Gilvibacter*
ASV29	Bacteroidetes	Bacteroidia	Chitinophagales	Chitinophagaceae	*Sediminibacterium*
ASV41	Proteobacteria	Gammaproteobacteria	Betaproteobacteriales	Burkholderiaceae	*Undibacterium*
ASV42	Proteobacteria	Gammaproteobacteria	Betaproteobacteriales	Burkholderiaceae	*Cupriavidus*
ASV43	Proteobacteria	Gammaproteobacteria	Betaproteobacteriales	Burkholderiaceae	*Paucibacter*
ASV47	Proteobacteria	Gammaproteobacteria	Betaproteobacteriales	Burkholderiaceae	*Variovorax*
ASV67	Proteobacteria	Gammaproteobacteria	Salinisphaerales	Solimonadaceae	unassigned
ASV83	Proteobacteria	Alphaproteobacteria	Sphingomonadales	Sphingomonadaceae	*Sphingomonas*
ASV93	Proteobacteria	Alphaproteobacteria	Caulobacterales	Hyphomonadaceae	unassigned
ASV98	Proteobacteria	Alphaproteobacteria	Rhizobiales	Beijerinckiaceae	*Methylobacterium*
ASV115	Proteobacteria	Alphaproteobacteria	Rhodobacterales	Rhodobacteraceae	*Marivita*
ASV118	Proteobacteria	Gammaproteobacteria	Alteromonadales	Marinobacteraceae	*Marinobacter*
ASV122	Proteobacteria	Alphaproteobacteria	Rhodobacterales	Rhodobacteraceae	unassigned

The data show that the variability between exponential and stationary phase samples was large, so that even abundant ASVs sometimes were not detected at all in the stationary phase, or vice versa. Similarly, the variability between biological replicates was also large, with ASVs not being detected at all in one of the three biological replicates (Supplementary Figure 3). One has to keep in mind that the bacterial DNA was a minor component of the bulk DNA that was dominated by the dinoflagellate. Moreover, extraction of DNA from dinoflagellates is hampered by their large amount of lipids and pigments. The PCR amplification efficiency of ASVs that had a mismatch to the primers used here might therefore strongly depend on the amount of PCR inhibitory compounds in the extracted DNA.

### Response of the microbiome to increased temperature

Increased temperature resulted in abundance changes of both the unique and the core community (Figure 4; Supplementary File 5), and these responses depended on the strain of *P. cordatum* and the ASV in question.

The bacterial community of strain CCMP1529 was the most stable one. There were no major temperature-related changes in ASV abundance except for ASV140 (*Sulfitobacter*) and ASV66 (*Oceanococcus*) being significantly more abundant at 20°C and ASV23 (*Gilvibacter*) and ASV63 (*Aestuaribacter*) being below detection already at 26°C. While *Gilvibacter* was a member of the core community, *Aestuaribacter* was unique for this dinoflagellate microbiome. Curiously, this dinoflagellate strain was not able to grow at 30°C.

In strain CCMP2811 the most abundant ASVs remained stable up to 30°C. The less abundant ASVs showed large variability. For example, three ASVs (ASV63, ASV109, and ASV72) were significantly more abundant at 20°C than at higher temperatures, while several rare ASVs increased in abundance at 30°C. Some of the ASVs that increased in abundance at 30°C were members of the core microbiome, e.g., ASV43 (*Paucibacter*). Some others that were significantly more abundant at 30°C were uniquely detected in this isolate, e.g., ASV120 and ASV144.

The microbial community associated with CCMP697 was the most diverse one and it responded most strongly to increased temperature. Some ASVs were significantly more abundant at 20°C than at the higher temperatures (ASV58, ASV34), with some dropping below detection (ASV118, ASV21, ASV121). ASV21 (*Lacinutrix*) is most notable because it was very highly abundant at 20°C, comprising 25 ± 6% of the total community in the free-living as well as the particle associated fraction, but dropped below detection at 26°C and 30°C. It was never found in any of the other *Prorocentrum* isolates, suggesting it might be specifically adapted to CCMP697 and a temperature of 20°C. A few ASVs had no significant change in abundance when grown at 20°C and 26°C, but were significantly less abundant at 30°C (11 ASV, Supplementary File 5). Finally, a large number of ASVs (33) were detected for the first time at 30°C (Figure 4), with ASV53 and ASV33 being significantly more abundant at 30°C compared to 20°C.

Strain CCMP698 was not able to grow at 30°C. It showed only few changes with increased temperature. There were two ASVs that significantly increased in abundance at 26°C, namely ASV141 (*Sedimentitalea*) which was detectable for the first time, and ASV27 (*Lewinella*) which became highly abundant at 26°C. Several ASVs were significantly less abundant at 26°C, e.g. ASV91, ASV52, ASV39, and ASV142 which were unique for this isolate, and ASV118 (*Marinobacter*) which belonged to the core microbiome.

### Response of the core microbiome to increased temperature

The core microbiome of the four strains of *P. cordatum* responded differently to increased temperature in each strain (Figure 5; Supplementary File 5). ASV23, a flavobacterium from the genus *Gilvibacter* was the most abundant ASV in our study and the dominant ASV in CCMP2811 at all temperatures. However, it significantly decreased in abundance in the other three isolates at higher temperatures, being below detection in CCMP1529 at 26°C and in CCMP697 at 30°C.

Similarly, ASV115, the second most abundant ASV in our study, a *Rhodobacteraceae* from the genus *Marivita,* was highly abundant at 20°C and 26°C in CCMP2811 and CCMP698. On the other hand, the abundance of this ASV was considerably lower in strains CCMP1529 and CCMP697, and decreased significantly at higher temperatures, being below detection in CCMP697 at 30°C. The other highly abundant core ASVs, namely ASV122 (*Rhodobacteraceae*) and ASV118 (*Marinobacter*) remained highly abundant in a temperature independent way in two of the isolates (CCMP1529, CCMP2811), however, significantly dropped in abundance at higher temperatures for strains CCMP697 and CCMP698. ASV93 (*Hyphomonadaceae*) significantly dropped in abundance at higher temperatures for all isolates except CCMP1529.

Interestingly, some of the core ASVs increased in abundance at higher temperatures. For example, the abundance of ASV43, a betaproteobacterium from the genus *Paucibacter*, increased in all four isolates with temperature, and particularly at 30°C in CCMP2811 and CCMP697.

### Growth of axenic and xenic cultures of *Prorocentrum cordatum* at different temperatures

We compared the growth of the four xenic cultures of *P. cordatum* with that of an isolate of this species which has been made axenic and has been maintained at NCMA as such since 1958, namely CCMP1329. All five strains were routinely cultivated at 20°C and shifted to the higher temperature by transferring 10% of a culture from late exponential phase to either 26°C or 30°C. The cultures were allowed to adapt to the new temperature for three to four transfers until growth of the dinoflagellate was determined by counting cells with a FACS cell counter. Growth was followed for 24–28 days, except for the 30°C cultures which could only be observed for 16 days because of logistic problems.

**Figure 6:**
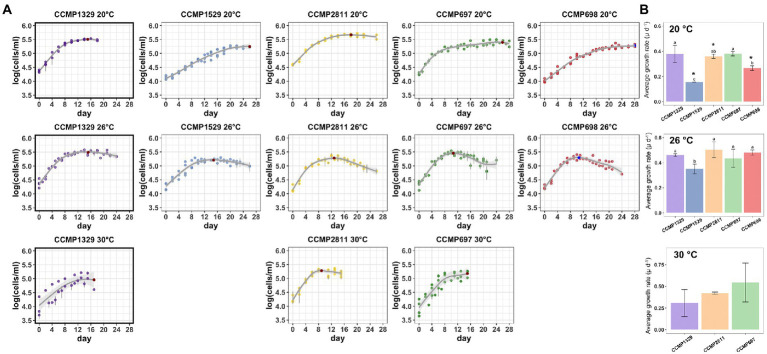
Growth of the five *Prorocentrum cordatum* strains at 20°C, 26°C, and 30°C. **(A)** Growth curves of each *Prorocentrum cordatum* strains at the different temperatures. Each point represents the average of triplicate cultures ± SD. Lines are from the best fitting GAM model quantifying the coupling between the logarithm of cell concentration as a function of time. **(B)** Growth rate barplots for each *P. cordatum* strain at each temperature. Values marked with different letters indicate statistically significant difference between the growth rate of strains for each temperature, while the (*) indicates significantly lower growth rate for that strain compared to 26°C (*p* < 0.05), there was no significant change in growth between 20°C and 30°C.

The results are shown in Figure 6 and Table 4. At 20°C, the doubling time of the axenic strain CCMP1329 was 1.83 days, similar to that of CCMP2811 (1.92 days) and CCMP697 (1.81 days), although the latter had been maintained at NCMA at 14°C and its known temperature range is 11°C–16°C. By contrast, CCMP1529 showed significantly slower growth at 20°C than the rest, with a doubling time of 2.56 days. Strain CCMP68 was significantly slower than CCMP1329 and CCMP697, with a doubling time of 4.44 days. This strain had also been maintained at NCMA at 14°C. So at 20°C the different strains showed more than twofold differences in growth rate. All of them were apparently able to adapt to 20°C, independent of their maintenance temperature at NCMA. None of them grew faster than the axenic strain CCMP1329.

**Table 4:** Growth parameters of the four xenic and one axenic strain of *Prorocentrum cordatum*.

	20°C			26°C			30°C		
Strain	Growth rate (μ^d-1^)	Doubling time (days)	Day of highest cell density	Growth rate (μ^d-1^)	Doubling time (days)	Day of highest cell density	Growth rate (μ^d-1^)	Doubling time (days)	Day of highest cell density
CCMP697	0.38	1.81	25.87	0.44	1.59	10.63	0.54	1.27	15.06
CCMP698	0.27	2.56	28	0.48	1.44	10.63	NA	NA	NA
CCMP1529	0.16	4.44	26	0.35	1.98	14.48	NA	NA	NA
CCMP2811	0.36	1.92	18.10	0.50	1.37	12.83	0.42	1.64	8.92
CCMP1329^*^	0.38	1.83	15.26	0.46	1.50	15.18	0.30	2.26	13.00

The cell concentrations (cell/ml) determined by flow cytometry for three independent growth curves at each temperature were fitted to a generalized additive model (GAM). Growth parameters were obtained as described in Materials and methods. Supplementary Figure 4 shows calculation of growth rate.

* Axenic. NA, strains CCMP1529 and CCMP698 were not able to grow at 30°C.

In addition to their different growth rates during exponential growth at 20°C, the five cultures showed striking differences during the transition to stationary phase. The axenic strain CCMP1329 shifted from exponential growth to zero growth within a few days, reaching its maximum cell density already after 15 days. By contrast, in the xenic cultures exponential growth was followed by a prolonged period of slow growth, until zero growth (stationary phase) was finally reached after 18, 26, 26 or 28 days, respectively.

At 26°C, strains CCMP1529, CCMP2811, and CCMP698 showed a significant increase in growth rate compared to 20°C, and the differences between them became smaller. CCMP1529 was again the slowest culture (doubling time 1.98 days), while the other four were similar (doubling time 1.50 days, 1.37 days, 1.59 days, and 1.44 days). CCMP2811 and CCMP698 grew slightly faster than the axenic strain. In contrast to their behavior at 20°C, the xenic cultures did not show a prolonged period of slow growth after their initial exponential growth. All of them reached their highest cell density after 11 to 15 days. Interestingly, all four xenic cultures showed a death phase after reaching their highest cell density. This death phase was strongest in CCMP698, where it resulted in a more than tenfold decrease in cell density in 13 days. The axenic strain CCMP1329 reached its highest cell density at a similar time as the xenic cultures, but a death phase was lacking. Only a small decrease in cell density—as expected in stationary phase—was observed.

At 30°C, only the axenic strain CCMP1329 and the xenic cultures CCMP2811 and CCMP697 were able to grow. The axenic strain showed slower growth than both xenic cultures. However, there was no significant difference between the three cultures.

## Discussion

We analyzed the bacterial communities from four different strains of the cosmopolitan dinoflagellate *P. cordatum*. These strains had been isolated from distant geographical locations with different environmental conditions, e.g., CCMP1529 near the coast of Ecuador and CCMP697 from the North Sea, Norway. They were maintained as live cultures under standardized cultivation conditions at the NCMA for many years, the longest being CCMP697 with 37 years, and the shortest CCMP2811 with 15 years. There are no cryocultures available for these strains (in contrast to the axenic strain CCMP1329), therefore we can very roughly estimate the lower limit for the number of generations that elapsed since deposition. If 1% of the culture were transferred every 4 weeks this would require 180 transfers in 15 years, or 444 transfers in 37 years. The average growth rate of the strains at 20°C was 0.29 μ⋅d^−1^ which translates to 8.7 generations per transfer, or 1,566 generations in CCMP2811 and 3,863 generations in CCMP697 from deposition till today. The microbiomes that we are studying here are therefore the result of a long-term adaptation and co-evolution of the initial seeding communities over hundreds of generations. We are studying a closed system where the heterotrophic bacterial community was entirely dependent on organic molecules excreted by the dinoflagellate. The L1-Si medium contained all nutrients required by the dinoflagellate, including the three vitamins for which *P. cordatum* and many bacteria are auxotrophic, and it was shared between the bacterial microbiome and the microalgal host.

### Host strain filtering of *Prorocentrum cordatum* microbiomes

The microbial communities from the four strains of *P. cordatum* showed large differences in composition and diversity. They were clearly separated on the NDMS plot, and this separation was only slightly modified by lifestyle (free living or particle attached) and temperature. The *P. cordatum* host strain was the strongest factor in determining the composition of the bacterial communities. Although we did not genotype the four strains of *P. cordatum* strains here, we assume that they represent different genomovars of the species *P. cordatum*.

In diatoms, the assembly of the phycosphere microbiome has been the subject of many studies, with the seeding community, genotype of host species or strain, and bacterial competition being the most important parameters affecting the resulting microbiome composition (Whittaker and Rynearson, 2017; Ajani et al., 2018; Behringer et al., 2018; Stock et al., 2022). Our finding is strikingly similar to a study of the diatom *Thalassiosira rotula* (Ahern et al., 2021) showing that the diatom host genotype was the strongest determinant of microbial community composition, which they termed genotype filtering (Ahern et al., 2021), while geographic location played a minor role. In our experiment, we are observing the outcome of co-adaptation between host and microbiome after many years of co-culturing under stable conditions, but such genotype filtering has nevertheless been observed after many years (Barreto Filho et al., 2021). In this respect, it is interesting to observe that alpha-diversity was largest in strain CCMP697. This strain has been the one with the longest time in the culture collection, almost four decades, suggesting that the community approached a climax state with maximum diversity. However, we cannot exclude that the original bacterial community deposited with the isolate showed the highest diversity already.

The four *P. cordatum* microbiomes were comprised of five main classes, Alphaproteobacteria, Bacteroidia (Flavobacteriaceae), Gammaproteobacteria, Phycisphaerae (found mainly in strain CCMP697), and Rhodothermia (identified only in strain CCMP698). These five groups have often been reported to be associated with dinoflagellates. Transcriptomic and metabolomic studies of a natural phytoplankton bloom (Teeling et al., 2016) and of bacteria co-cultured with a diatom (Ferrer-González et al., 2021) have demonstrated resource partitioning, with Alphaproteobacteria and Gammaproteobacteria using mainly low molecular weight compounds, while Bacteroidia degrade algal polysaccharides, potentially resulting in complementarity rather than competition between members of these classes.

Even though the four *P. cordatum* cultures were dominated by Alphaproteobacteria, Bacteroidia (family *Flavobacteriaceae*) and Gammaproteobacteria, the relative abundances of these three classes varied considerably between the microbial communities, because the dominant ASV of each dinoflagellate strain belonged to either of these classes. For example, 60.32% ± 4.8% of the bacterial community of strain CCMP1529 was comprised by ASV116 identified as *Marivita,* an Alphaproteobacterium. The most dominant ASV for strain CCMP2811 was ASV23 identified as *Gilvibacter*, which accounted for 40.4% ± 1.7% of its bacterial community and was the only ASV assigned to Bacteroidia (family *Flavobacteriaceae*) in this dinoflagellate. The most dominant ASV for strain CCMP697 was identified as *Lacinutrix* accounting for 25% ± 6% of its bacterial community and belonging to *Flavobacteriaceae* as well. These dominant ASVs were highly abundant in the respective dinoflagellate microbiome but had considerably lower abundance or were absent in the other strains. A similar pattern has been observed in the closely related *Prorocentrum lima* (Tarazona-Janampa et al., 2020). The data suggest that resource partitioning occurred not so much on the level of class, but rather on the level of ASVs. The large individuality of each algal microbiome likely reflects the complexity of compounds provided by the algal host and the multitude of taxonomically different communities that can adapt to each compound cocktail. Moreover, differences in the metabolism of the four dinoflagellates might also select different microbiomes.

### Core microbiome

Although the four *P. cordatum* cultures had clearly distinct bacterial communities, there were 14 ASVs that were shared among all four strains. They were all found in the PA community, so they are most likely attached to the dinoflagellate and passed over to the next generation with half of the growing theca. Half of the ASVs in the core microbiome had abundances below 1%, while the other 7 ASVs showed different abundances across dinoflagellate strains, from which ASV115 (*Marivita*) and ASV23 (*Gilvibacter*) had particularly high abundances. It is worth noticing that ASV23 was the only ASV in the dataset assigned to the genus *Gilvibacter*.

*Marivita* is an ecological generalist from the Roseobacter clade (Zheng et al., 2019) which has often been found in dinoflagellate phycosphere microbiomes (Cruz-López and Maske, 2016; Park et al., 2017; Zhou et al., 2021). *M.* c*ryptomonadis* and *M. litorea* are the first type species from the genus *Marivita*, which is currently comprised of five type species and six genomes (Zhou et al., 2021). The genome of *Marivita* sp. XM-24 was reconstructed from a xenic *Synechococcus* culture and showed the typical Roseobacter traits, including potential for aerobic anoxygenic photosynthesis, DMSP demethylation, PHA polyhydroxy acid metabolism, inorganic sulfur oxidation, oxidation of carbon monoxide, and transporters for uptake of nitrogen and phosphorus compounds (Zheng et al., 2019). In *Protoceratium reticulatum*, which produces large amounts of DMSP, *Marivita* was the most abundant genus, suggesting that the amount of DMSP produced by a dinoflagellate might correlate with the abundance of *Marivita* in its phycosphere microbiome (Lin et al., 2021).

*Gilvibacter* is a genus of *Flavobacteriaceae* with the type species *G. sediminis* isolated from marine sediment in Okinawa (Khan et al., 2007). *G. sediminis* comprised 20% of the endosymbiotic microflora of the toxic dinoflagellate *Pyrodinium bahamense* (Onda et al., 2015). *Gilvibacter* ASVs were also found on marine surfaces (Papadatou et al., 2021).

Interestingly, four of the 14 ASVs comprising the core community belonged to the Gammaproteobacteria family of *Burkholderiaceae*. It was also the only family that had several ASVs within the core microbiota. The members of this family are highly versatile, interacting with animals, humans, plants and fungi (Bontemps et al., 2010; Hoffman and Arnold, 2010). They have been associated with micro -and macro-algae bacterial communities (Hurtado-McCormick et al., 2019; Steichen et al., 2020; Qu et al., 2021; Yu et al., 2022). These bacteria can fix N2 providing nutrients for the algae host (Bontemps et al., 2010; Gyaneshwar et al., 2011). *Marinobacter*, another member of the gammaproteobacterial core community, produces the siderophore vibrioferrin (VF), which is supplied to the microalgae as a bioavailable form of iron (Amin et al., 2009; Yarimizu et al., 2018).

Within the Alphaproteobacteria there was a *Methylobacterium* ASV in the core bacterial community. It has been discovered that the microalgae *Chlamydomonas reinhardtii* is able to grow efficiently on several inorganic nitrogen sources as well as on many amino acids, thanks to a mutualistic exchange of carbon and nitrogen compounds with *Methylobacterium* spp. (Calatrava et al., 2018). Finally, ASVs of *Hyphomonadaceae* and *Sphingomonas* were also found in the core community. Volatile compounds of these two bacteria, particularly indole and CO_2_, have been reported to significantly improve growth of the microalgae *Chlorella vulgaris* (Cho et al., 2019).

### Effect of temperature on the bacterial communities of *Prorocentrum cordatum*

Here we analyzed how temperature affected the phycosphere microbiome of the four *P. cordatum* strains. There was no significant change in alpha diversity of the bacterial communities when growing the cultures at 26°C or 30°C compared to 20°C. Temperature had a weak although significant influence on beta-diversity, with R^2^ between 0.217 (CCMP1529) and 0.327 (CCMP697; Table 2). However, there were strain specific changes in the composition of the bacterial communities. Each dinoflagellate culture had ASVs found uniquely at each temperature.

The data suggest that the temperature specific ASVs were present in the phycosphere microbiomes at low abundance and grew to higher densities at higher temperatures. For example, the gammaproteobacterium *Paucibacter* was ubiquitous in all *P. cordatum* cultures with an abundance below 1% at lower temperatures, but became the most abundant ASV in strain CCMP697 at 30°C. Such differential abundances could be caused by different temperature adaptations of the respective bacterial ASVs, but since 30°C is the optimal growth temperature for most marine bacteria, for example *D. shibae* (Biebl et al., 2005) this seems to be unlikely. Most probably, the metabolism of the dinoflagellate changed with temperature, resulting in different amounts and types of excreted metabolites. The low abundant ASVs of the cultures might thus provide a source of genomic variability that can respond to changes in the compound cocktail provided by the dinoflagellate, in a similar way as the rare biosphere in the ocean provides a pool of low abundant bacterial taxa that can quickly respond to environmental changes (Jousset et al., 2017; Ziegler et al., 2018; Pascoal et al., 2021).

As would be expected, we found that the two dinoflagellate cultures that had been maintained at 20°C at the culture collection (CCMP1529 and CCMP 2811) showed relative few changes in the abundance of their ASVs at increased temperature, thus their phycosphere microbiomes were stable, in comparison with the two cultures that had been maintained at 14°C at the culture collection (CCMP697 and CCMP698). Interestingly, the cold-adapted CCMP697 showed the largest number of differentially abundant ASVs at higher temperature. It also had the largest number of ASVs that were found only at 30°C, and in spite of being cold-adapted it was able to grow at 30°C. This finding might suggest that the shift in the composition of its bacterial community may have supported its adaptation to higher temperatures (Osman et al., 2020; Voolstra and Ziegler, 2020; Savary et al., 2021), but further studies would be needed to prove this.

### Growth with and without bacteria

The role of temperature for growth of *P. cordatum* has been studied in depth (Grzebyk and Berland, 1996; Heil et al., 2005; Khanaychenko et al., 2019), but the influence of the associated microbiome has not been considered previously. Here we determined the growth rates of the four xenic cultures of *P. cordatum* described above at 20°C, 26°C, and 30°C and compared them to that of the axenic *P. cordatum* CCMP1329. The growth rates μ⋅d^−1^ measured here for the various cultures of *P. cordatum* were between 0.16 and 0.54. Values reported in the literature are between 0.6 and 2.4 under high irradiance, 206 μmol quanta m^−2^ s^−1^ and natural light > 172 μmol quanta m^−2^ s^−1^, respectively (Khanaychenko et al., 2019). Thus, our growth rates are at the lower end of those reported in the literature, most probably due to the low light intensity used in our experiments (about 40 μmol quanta m^−2^ s^−1^) and due to the fact, that some strains were originally isolated from cold ocean regions, namely CCMP697 and CCMP698. At 20°C and low light intensity (17.2 μmol quanta m^−2^ s^−1^) a growth rate of *P. cordatum* of 0.41 d^−1^ was previously reported (Solomonova and Mukhanov, 2015), similar to that of our five cultures. Here we found clear differences between the growth rates of five different cultures of *P. cordatum* at 20°C which may be related to genetic differences between the isolates and the composition of their associated microbial communities.

Ecological niche modeling showed *P. cordatum* to be extremely tolerant to temperatures up to 28°C (Goncharenko et al., 2021). Interestingly, here we found that the axenic strain was able to grow at 30°C, while two of the xenic cultures were not. Genetic differences between the host strains or differences in the strains’ microbiota could contribute to these differences.

The two striking observations regarding the growth of CCMP1329 in comparison with the four xenic cultures were (1) the slow transition from exponential to stationary growth at 20°C and (2) the death phase observed at 26°C. Both observations were made for all four xenic cultures, i.e. they were independent of the actual composition of the associated microbial communities which were quite different and also responded to increased temperature differently, as shown in detail above.

Like many dinoflagellates, *P. cordatum* is mixotrophic (Heil et al., 2005; Khanaychenko et al., 2019). The dinoflagellate can take up bacteria (and other microalgae) and thus obtains nitrogen, phosphorus and amino acids and this shift from autotrophic to mixotrophic growth occurs on a large scale in the ocean (Cohen et al., 2021). Our axenic strain CCMP1329 growing in L1 mineral medium was entirely dependent on its own photosynthesis; all cellular components including all amino acids had to be synthesized. Thus, the metabolism of CCMP1329 was phototrophic, while that of the four xenic strains was mixotrophic and thus most probably differed from that of the axenic culture in fundamental aspects. We suggest that the slow transition from exponential to stationary growth in the xenic cultures was due to the accompanying microbiome, because it occurred in all xenic cultures independently of the composition of the associated microbiome. This hypothesis would have to be proven by inoculation of the axenic CCMP1329 with the microbiota from the xenic strains.

Similarly, we suggest that the death phase that was observed in all xenic cultures, but not in CCMP1329 at 26°Cwas most likely due to the accompanying microbial community, and again this hypothesis would need to be proven.

The way the microbial community might induce a death phase of the algae could be algicidal compounds (Meyer et al., 2017) or competition for shared nutrients. For example, *Pseudoalteromonas haloplanktis* AFMB-08041 lysed cells of *P. cordatum* within 5 days, while other microalgae were not affected (Kim et al., 2009), and *Alteromonas* strain FDHY-03 has exhibited high algicidal activity against the closely related dinoflagellate *P. donghaiense* (Shi et al., 2018). We did find an *Alteromonas* ASV within the bacterial community of strain CCMP697. However, in our experiment, algicidal bacteria are unlikely to have caused the death phase. Given the large differences in the community composition of the four xenic cultures, large differences in the death phase would have been expected depending on the presence or absence of an algicidal strain.

Competition for shared nutrients is another possibility. Eukaryotic marine microalgae are auxotrophic for one, two or all three of the vitamins B1, B7 and B12 (thiamine, biotin, and cobalamin; Croft et al., 2006) which are provided by the microbiome in nature (Croft et al., 2005). Most of the marine roseobacters can synthesize B12 *de novo* which may be one of the reasons why they are routinely found in the algal phycosphere and also Gammaproteobacteria can synthesize vitamins B1 and B12 (Paerl et al., 2015; Joglar et al., 2021). The culture medium in our experimental cultures initially contained all three vitamins. Some bacteria, like Bacteroidia require vitamins too (Cooper et al., 2019; Shelton et al., 2019; Joglar et al., 2021). We therefore expect competition for vitamins in the xenic cultures particularly at the higher growth rates observed at 26°C.

## Conclusion

Our study of the phycosphere communities of four strains of *P. cordatum* that had been isolated from distant geographical locations and maintained at the NCMA culture collection for hundreds of generations showed large individuality. Host strain was the major determinant of community composition, while growth phase, temperature, and lifestyle (free-living or particle attached) had only a modulating influence. We found a core community of 14 ASVs which were present in all four phycosphere microbiomes and may have been optimally adapted to the specific exometabolites of *P. cordatum*. The phycosphere communities responded to temperature stress by changes in abundance of both the core and the unique ASVs. The role of the bacteria for growth of the dinoflagellate was ambiguous. Three observations regarding the xenic cultures, namely a prolonged transition to stationary phase at 20°C, a death phase at 26°C, and lack of two xenic cultures to grow at 30°C, led us to hypothesize competition between dinoflagellate and bacteria for limiting nutrients, including essential vitamins, which would need to be proven experimentally.

## Data availability statement

The raw data generated for this study can be found in the National Center of Biotechnology Information (NCBI) repository under the BioProject accession number: PRJNA831420. The datasets analyzed in the study are present in Supplementary material.

## Author contributions

IW-D and SS-G designed the study and wrote the manuscript. SS-G and HW did the experimental work. SS-G carried out the data analysis. All authors contributed to the article and approved the submitted version.

## Funding

This work was funded by the Deutsche Forschungsgemeinschaft within the Collaborative Research Center TRR51 *Roseobacter*.

## Conflict of interest

The authors declare that the research was conducted in the absence of any commercial or financial relationships that could be construed as a potential conflict of interest.

## Publisher’s note

All claims expressed in this article are solely those of the authors and do not necessarily represent those of their affiliated organizations, or those of the publisher, the editors and the reviewers. Any product that may be evaluated in this article, or claim that may be made by its manufacturer, is not guaranteed or endorsed by the publisher.

## Supplementary material

The Supplementary material for this article can be found online at: https://www.frontiersin.org/articles/10.3389/fmicb.2022.952238/full#supplementary-material
