# Supplementary material for: The microbiome of the dinoflagellate Prorocentrum cordatum in laboratory culture and its changes at higher temperatures
Source: Front Microbiol. 2022 Sep 28;13:952238. doi: 10.3389/fmicb.2022.952238 (PMC9555710; doi:10.3389/fmicb.2022.952238)
Supplement: Supplementary file 2 [file Data_Sheet_1.DOCX]

Supplementary Figures

##
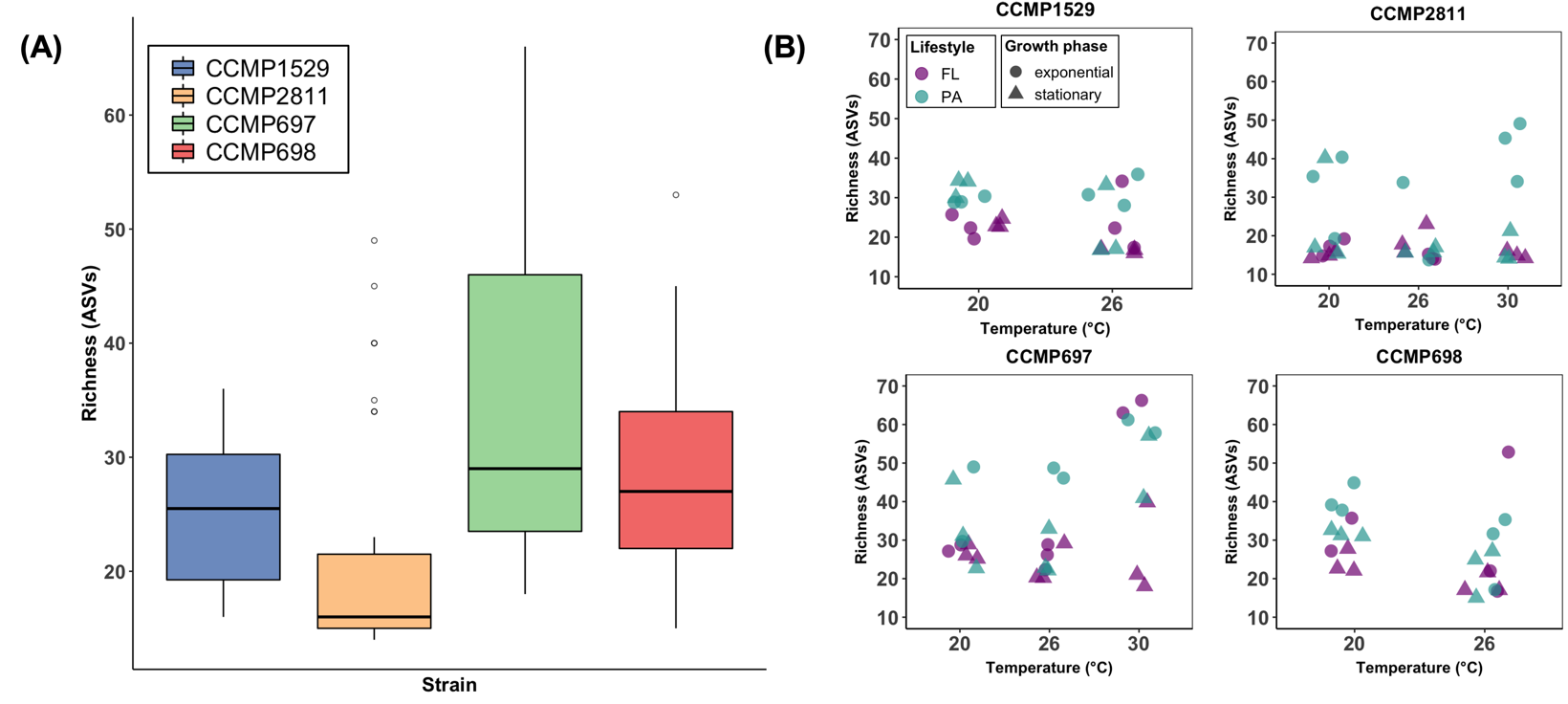
Supplementary Figures

**Supplementary Figure 1.** Analysis of Alpha diversity of bacterial communities of *P. cordatum* measured by mean richness of ASVs. **(A)** Total richness of the bacterial communities. All samples were grouped by strain and are indicated by color. The solid black lines indicate medians, and the lower and upper bounds of the box represent the 25 and 75% quartiles. Outliers are indicated as open circles and represent samples falling outside the 10 and 90% quartiles. **(B)** Richness diversity between temperature (x-axis), lifestyles (color) and growth phase (shape) was analyzed for the bacterial communities of each *P. cordatum* strain.


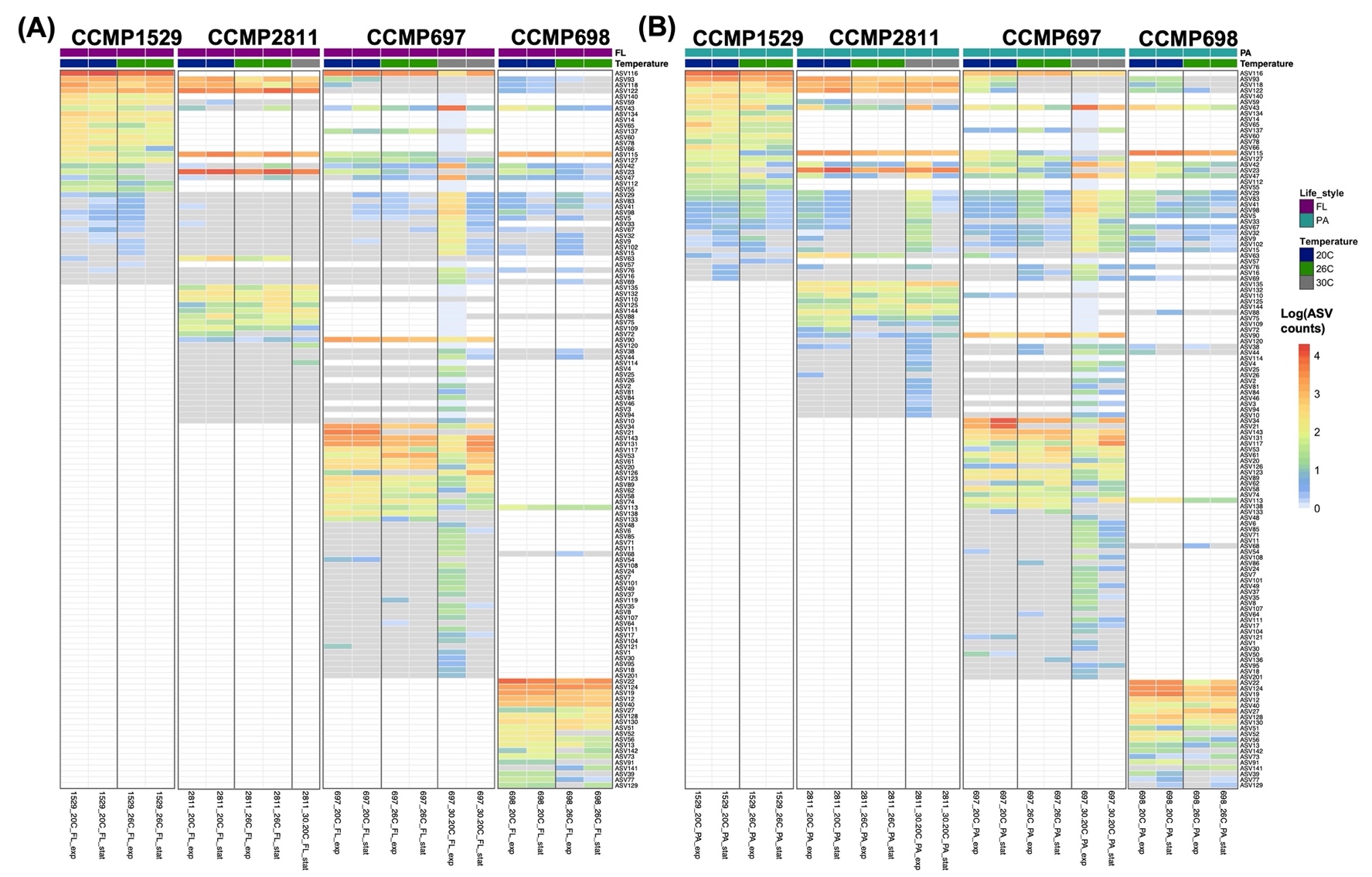
**Supplementary Figure 2.** Abundance of all ASVs detected in four xenic strains of *P. cordatum*. at different phases and temperatures for **(A)** the FL community and **(B)** PA community. The heatmaps show ASVs based on their abundances, expressed as log(1 + x). After AMCOM-II preprocessing, white squares correspond to “structural zeros” (lack of ASVs) and grey squares to “sampling zeros” (zeros due to abundance below detection limit /sequencing depth) (Kaul *et al.*, 2017). The average AVS abundance was calculated from two or three biological replicates for each strain at each condition, where the colors in the first row indicate lifestyle and the ones in the second-row temperature.


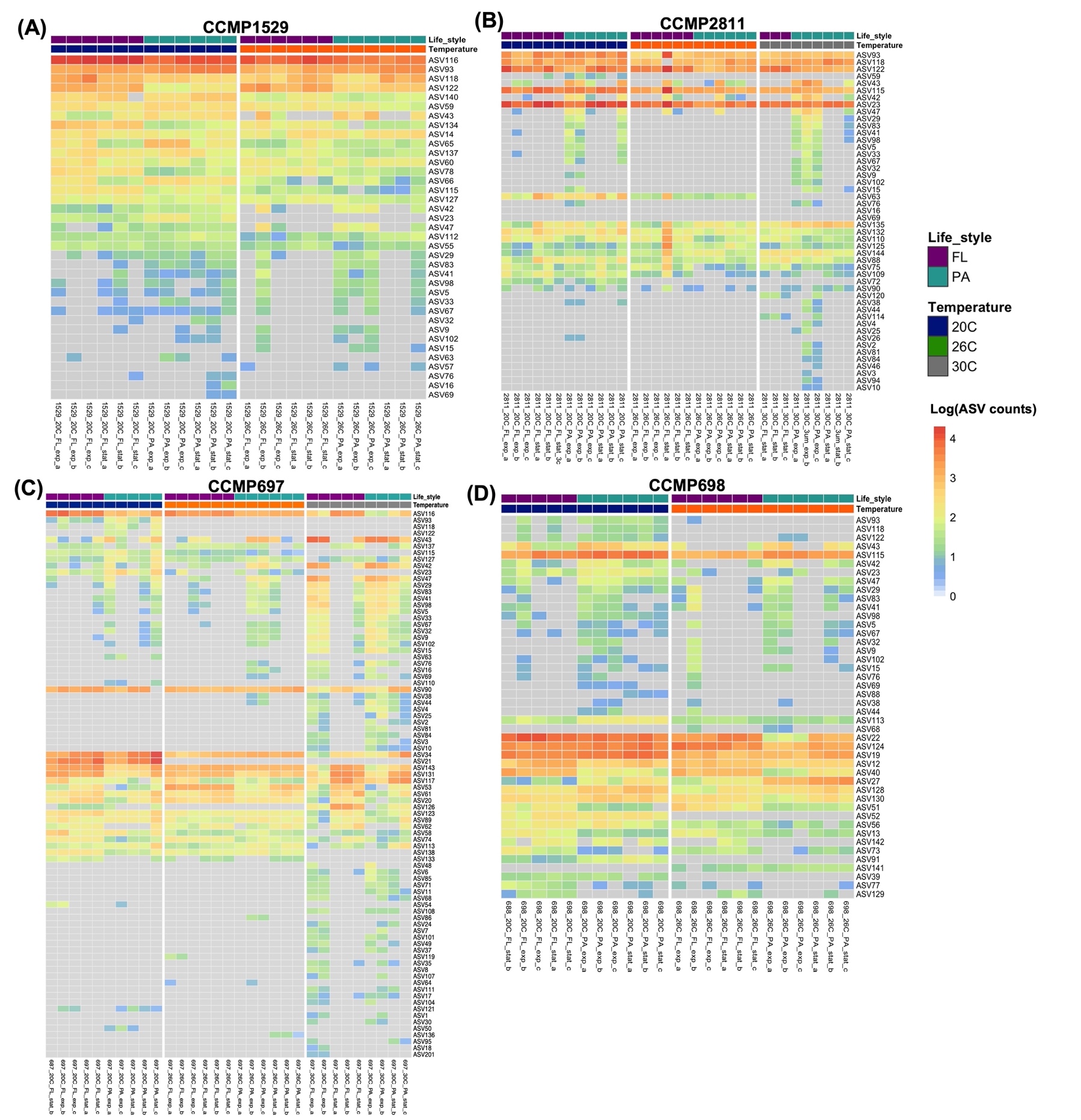
**Supplementary Figure 3.** Composition of the bacterial communities of CCMP697, CCMP698, CCMP1529 and CCMP2811. Heatmaps show ASV abundances of biological replicates for each strain at each condition **(A-D)**, where the colors in the first row indicate lifestyle and the ones in the second row temperature. See Figure 4 for details of calculation.


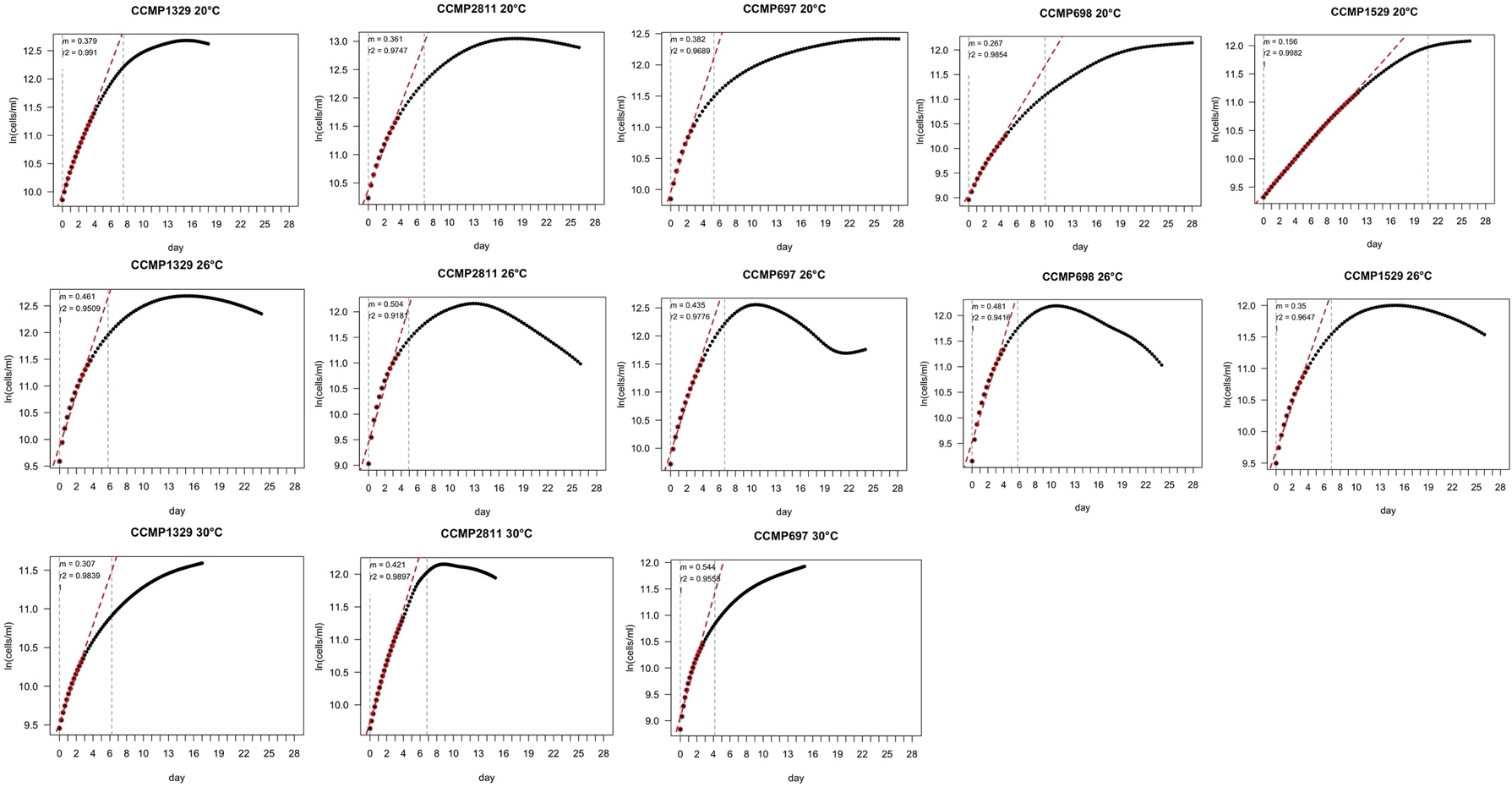


**Supplementary Figure 4.** Determination of growth rates for the different *P. cordatum* strains at 20 °C, 26 °C and 30 °C**.** The output of the GAM models obtained from the growth curves for each strain (refer to Materials and Methods) is plotted in black dots on a log-scale. The points outlined in red represent the interval which had the maximum slope. Each point is considered part of the exponential growth phase if its residual off the line of best fit is less than 5%. The dashed red line is the line of best fit (R^2^> 0.9) obtained by the linear modeling function lm(). The vertical light blue dashed lines represent the exponential growth phase.
